# Cryopreservation and transplantation of ovarian tissue as post-cancer tissue therapy and an activator of oogenesis

**DOI:** 10.1186/s13048-025-01680-9

**Published:** 2025-06-10

**Authors:** Volodimir Isachenko, Bernd Morgenstern, Plamen Todorov, Evgenia Isachenko, Frank Nawroth, Maria Quassdorff, Mahmoud Salama, Nina Mallmann-Gottschalk, Markus Merzenich, Christine Skala, Gohar Rahimi

**Affiliations:** 1 Department of Obstetrics and Gynecology, Medical Faculty, Cologne University, Cologne, Germany; 2 Institute of Biology and Immunology of Reproduction of Bulgarian Academy of Sciences (BAS), Sofia, Bulgaria; 3 AMEDES Facharzt-Zentrum für Kinderwunsch, pränatale Medizin, Endokrinologie und Osteologie GmbH, Hamburg, Germany; 4 Department of Obstetrics, Gynecology and Reproductive Biology, College of Human Medicine, Michigan State University, Michigan, USA; 5 Department of Obstetrics and Gynecology, Medical Faculty, Duisburg-Essen University, Essen, Germany; 6 Medizinisches Versorgungszentrum AMEDES für IVF- und Pränatalmedizin in Köln GmbH, Cologne, Germany

**Keywords:** Ovarian tissue cryopreservation, GV oocyte maturation, Hippo signaling disruption, Hypoxia, Intracellular Ca²⁺ increase, Osmotic disruption of cellular membranes, Reactive oxygen species (ROS) generation, Lipid peroxidation

## Abstract

In a recent publication (Reprod. Biomed. Online, 2024) it was presented point of view that, for post-cancer patients, in vitro fertilization (IVF) is a more effective method than ovarian tissue cryopreservation (OTC). In their commentary, Andersen et al. (Reprod. Biomed. Online, 2024) present nine distinct arguments advocating for the use of OTC. We fully agree with all these points. In support of the clinical application of the OTC procedure, we introduce two additional arguments. First, the transplantation of cryopreserved ovarian tissue can be considered as a form of tissue therapy. Moreover, OTC inherently serves as an activator of oogenesis. Second, during tissue dissection prior to OTC, a significant number of germinal vesicle (GV) oocytes can be retrieved, matured to the metaphase II (MII) stage, cryopreserved, and later used in IVF procedures.

## Ovarian tissue of cancer patients: IVF program vs. cryopreservation

Our ovarian tissue cryobank was established in 2001. We read with great interest the publication by Macklon and De Vos [1] on methods for treating infertility in cancer patients. The authors compare two approaches for restoring reproductive function following anti-cancer therapy: the IVF program (hormonal induction of superovulation, oocyte fertilization, and embryo transfer) and ovarian tissue cryopreservation (OTC) with transplantation after cancer treatment. Their opinion suggests that, for post-cancer patients, the IVF program is a more effective method than ovarian tissue cryopreservation [1].

We also read with great interest the commentary by Andersen et al. [2], in which the authors provide nine medical, biological, and organizational arguments demonstrating the advantages of OTC and its necessity in the treatment of post-cancer patients. We fully agree with Andersen et al. [2], who highlight the benefits of OTC compared to the IVF program. The essence of their conclusion can be summarized as follows: while the IVF program preserves 10–20 oocytes, OTC preserves thousands.

We would like to add two arguments to Andersen et al. nine points [2] in favor of ovarian tissue cryopreservation (Table 1).

**Table 1:** Post-cancer patients: IVF program vs. Cryopreservation of ovarian tissue

Respective features of IVF-program	Respective features of ovarian tissue cryopreservation (OTC)-program
Mandatory hormonal induction of folliculogenesis, also in case of cancer with hormone-positive receptors	Natural (spontaneous) folliculogenesis without hormonal treatment
Transfer of about ten embryos, stretched out over months	Transfer of dozens of embryos, stretched out over years
Post-cancer restoration of sexual cycle by injection of hormones in case of cancer with hormone-positive receptors	Post-cancer natural restoration of sexual cycle without injection of hormones
No tissue therapy	Tissue therapy

## Ovarian tissue re-transplantation as an effective tissue therapy

First, ovarian tissue cryopreservation (OTC) should be regarded as an effective tissue therapy, wherein a patient receives a transplant of “young” ovarian tissue with increased viability. A notable example is a patient with diminished ovarian reserve who successfully gave birth to a healthy child after four years of pre-menopause following ovarian tissue transplantation [3].

Briefly, at age 33, this patient was diagnosed with invasive ductal breast cancer (MammaCa pT1b pN0 (073 sn) M0 V0 L0 G2 Pn0 R0, 4 × 9 mm). The tumour was resected. Chemotherapy (3 weeks primary anti-hormonal therapy with Tamoxifen, 8 cycles Nab-Paclitaxel and 4 cycles Epirubicin/ Cyclophosphamide) was administered. Before beginning of chemotherapy it was detected the pre-menopausal status. Before cryopreservation of ovarian tissue (Patient W. was 33 y. o., had body length 165 cm and weight 58 kg, BMI = 22) and beginning of anticancer treatment it was detected the idiopathic ovarian insufficiency (low ovarian reserve of unknown cause) of Patient W.: 56 IU/l FSH, 8 ng/l β-estradiol, < 1.1 ng/ml anti-Müllerian hormone. The number of follicles immediately after thawing and in vitro culture did not increase, as only one primordial follicle was found in 5 mm³ of tissue. However, 37 days after ovarian tissue re-transplantation, the patient’s menstrual cycle resumed. Three months post-transplantation, she became spontaneously pregnant and later delivered a healthy baby [3].

## Oocytes as a “by-product” of pre-freezing preparation of ovarian tissue

Second, the concept of our cryobank [4] includes the collection of Germinal Vesicle (GV) oocytes, their maturation to Metaphase II (MII) stage [5], and their cryopreservation (vitrification). These oocytes represent a valuable “by-product” obtained during the preparation of ovarian tissue fragments for freezing (cutting into fragments and partially removing the medulla).

Why should (GV) oocytes be collected before ovarian tissue freezing? If follicles are not retrieved via aspiration and GV oocytes are not cryopreserved before freezing, all antral follicles will be destroyed during tissue cryopreservation. This is because, after thawing, follicular development and GV oocyte formation occur only from preantral follicles. Follicles with fully formed, fluid-filled antra have zero cryo-resistance and do not survive the freezing process [5].

**Fig. 1:**
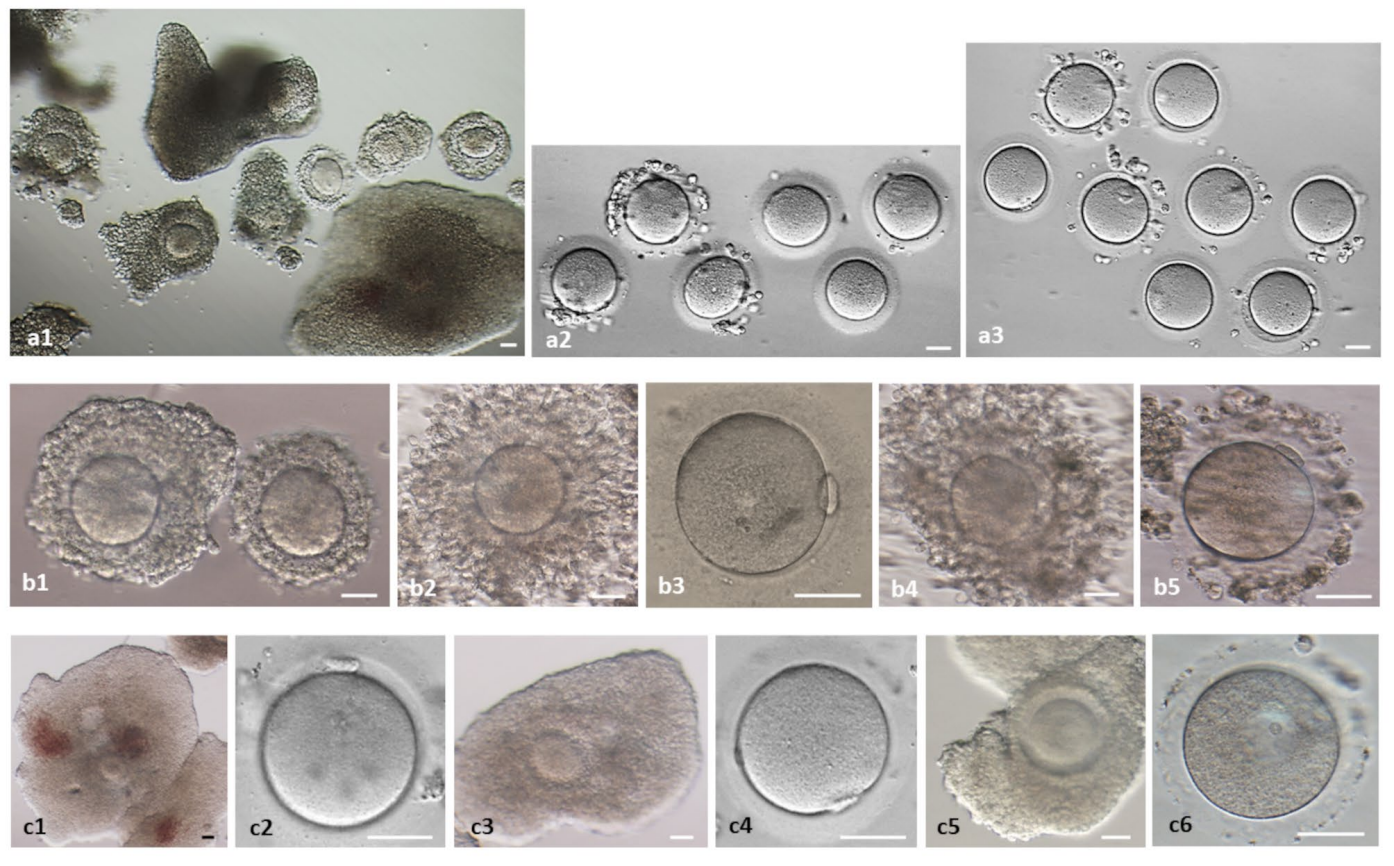
Preparation of ovarian tissue (dissection) before cryopreservation: oocyte-cumulus complexes (OCC) and in vitro maturation of germinal vesicle (GV)-oocytes to Metaphase II (MII)-stage. **a1-3** Patient M., 31 years old, breast cancer, 14 retrieved OCCs; **a1** OCC after preparation of ovarian tissue for freezing; **a2** 28 h maturation, 6 not matured (GV (left)- and MI (rest)-oocytes); **a3** 8 matured MII-oocytes. **b1-5** Patient K., 25 years old, Hodgkin’s lymphoma, 33 retrieved OCCs, 21 matured to MII stage; **b1** 2 OCC after preparation of ovarian tissue for freezing; **b2**,**3** left OCC after 28 h maturation and MII-oocyte; **b4**,**5** right OCC after 28 h maturation and MII-oocyte. **c1-6** Patient S., 34 years old, uterine carcinoma, 18 retrieved OCCs, 8 matured to MII stage; **c1-2** OCC after preparation of ovarian tissue for freezing and MII-oocyte after 28 h maturation of this OCC; **c3-4** OCC after preparation of ovarian tissue for freezing and MII-oocyte after 28 h maturation of this OCC; **c5-6** OCC after preparation of ovarian tissue for freezing and GV-oocyte after 28 h maturation of this OCC. Bar = 100 μm

Up to 20 high-quality GV oocytes (oocyte-cumulus complexes) can be obtained per patient (Fig. 1).

These GV oocytes can be matured to the MII stage [6]. Briefly, oocyte-cumulus complexes (OCCs) were washed three times with IVF medium (Fujifilm Irvine Scientific, Irvine, CA, USA) and transferred to maturation medium. Maturation was performed in the same IVF medium supplemented with 3 IU/mL recombinant FSH (Gonal F, Serono, Unterschleissheim, Germany), 3 IU/mL human chorionic gonadotropin (hCG) (Pregnesin, Serono), 1 µg/mL 17β-estradiol (Sigma-Aldrich, Munich, Germany), insulin–transferrin–selenium (10 µg/mL, 5.5 µg/mL, and 6.7 ng/mL, respectively; Gibco Life Technologies Ltd, Berlin, Germany), and 10% late follicular phase patient serum. Oocytes were cultured at 37 °C in 6% CO₂ for 28 h. MII oocytes were then cryopreserved (vitrified) for subsequent fertilization and embryo development. Published in Isachenko et al. [3] Fig. 4a illustrates the complete set of OCCs after extended maturation. In this study, we show (Fig. 1) individual complexes and oocytes both immediately after ovarian tissue dissection and after 28 h of in vitro culture.

## Cryopreservation of ovarian tissue as an activator of oogenesis

It is well known that primordial follicles in premature ovarian failure (POF) patients fail to activate normally, preventing the retrieval of mature oocytes for IVF [7, 8]. However, in vitro activation (IVA) of primordial follicles enables their development, presenting a new potential treatment for such patients [9].

We propose a hypothesis to explain the effectiveness of ovarian tissue re-transplantation in patients with a very low ovarian reserve. During cell cryopreservation, at least five negative effects occur: hypoxia, intracellular Ca²⁺ increase, osmotic disruption of cellular membranes, reactive oxygen species (ROS) generation, and lipid peroxidation. The Hippo signaling pathway is an evolutionarily conserved mechanism that regulates organ size by controlling cell proliferation, apoptosis, and stem cell self-renewal. Each of these “cryopreservation factors”, mentioned above can lead to disruption of ovarian Hippo signaling [10], which may, paradoxically, result in follicular activation. We suggest that the cryopreservation procedure itself may serve as an in vitro activation mechanism for follicles [3].

## Risk at transplantion of cryopreserved ovarian tissue contaminated with cancer cells

When comparing two methods of post-cancer reproductive restoration in patients, it would be completely inappropriate to ignore the risk of transplanting ovarian tissue contaminated with cancer cells. This risk is absent in IVF procedures. The issue becomes particularly relevant given the established fact that cancer cell malignancy increases after cryopreservation.

Some studies [11, 12] were conducted on two breast cancer cell lines, ZR-75-1 and MDA-MB-231. After cryopreservation (freezing and thawing) and in vitro culture, it was found that ZR-75-1 cells underwent morphological changes, shifting from typical grape-like clusters to a fibroblast-like or spindle-shaped form, detaching from neighboring cell clusters. The formation of filopodia and lamellipodia was observed. These morphological changes were associated with increased cell motility [11, 12].

Cryopreservation was noted to enhance the migratory ability and invasiveness of cancer cells. The data showed that the invasive capacity of the cells significantly increased after cryopreservation. The number of migrated and invaded cells after 72 h (ZR-75-1) and 8 h (MDA-MB-231) of in vitro culture was significantly higher after cryopreservation [11, 12].

GATA3 gene expression was significantly reduced in ZR-75-1 cells after cryopreservation compared to pre-treatment levels. Our data demonstrated that cryopreservation led to the loss of intercellular adhesion in cancer cells. Cryopreservation enhanced cell motility by increasing of structural proteins vimentin and F-actin levels. The expression of these two cytoskeletal proteins was significantly elevated in cells after cryopreservation compared to pre-cryopreservation levels. It was also observed a decrease in gene GATA3 and protein E-cadherin expression in cryopreserved cells. Our findings suggest that cryopreservation induces greater migration and invasion capabilities in breast cancer cells by increasing vimentin and F-actin expression and reorganizing intermediate filaments and microfilaments [11, 12].

In short, the cryopreservation procedure increases the malignancy and metastatic potential of breast cancer cells.

How can the information presented in this publication benefit clinical practice or patients? In our opinion, clinical specialists (reproductive specialists and oncologists) should consider the following when making decisions about reproductive function restoration plans for patients with metastatic cancer:

1. Conduct additional ovarian tissue studies, including: Histopathological Examination, Molecular and Genetic Testing, Flow Cytometry using fluorescently labeled antibodies to detect surface markers, commonly used in leukemia and lymphoma detection, Imaging-Based Methods.
2. Carefully evaluate the possibility of replacing ovarian tissue cryopreservation with IVF procedures.
3. Utilize the method of creating and transplantation of artificial ovary from a thawed fragment of ovarian tissue.

## Data Availability

No datasets were generated or analysed during the current study.
